# Subcortical functional connectivity and its association with walking performance following deployment related mild TBI

**DOI:** 10.3389/fneur.2023.1276437

**Published:** 2023-12-14

**Authors:** Mary R. Newsome, Sarah L. Martindale, Nicholas Davenport, Emily L. Dennis, Marlene Diaz, Carrie Esopenko, Cooper Hodges, George R. Jackson, Qisheng Liu, Kimbra Kenney, Andrew R. Mayer, Jared A. Rowland, Randall S. Scheibel, Joel L. Steinberg, Brian A. Taylor, David F. Tate, J. Kent Werner, William C. Walker, Elisabeth A. Wilde

**Affiliations:** ^1^Research Service Line, George E. Wahlen VA Medical Center, Salt Lake City, UT, United States; ^2^Traumatic Brain Injury and Concussion Center, Department of Neurology, University of Utah, Salt Lake City, UT, United States; ^3^H. Ben Taub Department of Physical Medicine and Rehabilitation, Baylor College of Medicine, Houston, TX, United States; ^4^Research and Academic Affairs Service Line, W. G. (Bill) Hefner VA Healthcare System, Salisbury, NC, United States; ^5^Veterans Integrated Service Networks (VISN)-6 Mid-Atlantic Mental Illness, Research Education and Clinical Center (MIRECC), Durham, NC, United States; ^6^Department of Physiology and Pharmacology, Wake Forest School of Medicine, Winston-Salem, NC, United States; ^7^Research Service Line, Minneapolis VA Health Care System, Minneapolis, MN, United States; ^8^Department of Psychiatry and Behavioral Sciences, University of Minnesota, Minneapolis, MN, United States; ^9^Research Service Line, Michael E. DeBakey Veterans Affairs Medical Center, Houston, TX, United States; ^10^Department of Rehabilitation and Human Performance, Icahn School of Medicine, New York, NY, United States; ^11^Department of Psychology, Brigham Young University, Provo, UT, United States; ^12^Parkinson's Disease Research, Education and Clinical Center (PADRECC), Michael E. DeBakey VA Medical Center, Houston, TX, United States; ^13^Department of Neurology, Baylor College of Medicine, Houston, TX, United States; ^14^Center for Translational Research on Inflammatory Diseases (CTRID), Baylor College of Medicine, Houston, TX, United States; ^15^Department of Neurology, Uniform Services University, Bethesda, MD, United States; ^16^The Mind Research Network/Lovelace Biomedical and Environmental Research Institute, Albuquerque, NM, United States; ^17^Departments of Psychiatry and Behavioral Sciences, Psychology and Neurology, University of New Mexico, Albuquerque, NM, United States; ^18^Department of Neurobiology and Anatomy, Wake Forest School of Medicine, Winston-Salem, NC, United States; ^19^Department of Psychiatry, Institute for Drug and Alcohol Studies, Virginia Commonwealth University, Richmond, VA, United States; ^20^Department of Imaging Physics, The University of Texas MD Anderson Cancer Center, Houston, TX, United States; ^21^National Intrepid Center of Excellence, Walter Reed National Military Medical Center, Bethesda, MD, United States; ^22^Department of Physical Medicine and Rehabilitation, Virginia Commonwealth University, Richmond, VA, United States

**Keywords:** basal ganglia, movement disorders, globus pallidus, functional connectivity, traumatic brain injury (TBI), service members and veterans, deployment (military), subcortical

## Abstract

**Introduction:**

The relation between traumatic brain injury (TBI), its acute and chronic symptoms, and the potential for remote neurodegenerative disease is a priority for military research. Structural and functional connectivity (FC) of the basal ganglia, involved in motor tasks such as walking, are altered in some samples of Service Members and Veterans with TBI, but any behavioral implications are unclear and could further depend on the context in which the TBI occurred.

**Methods:**

In this study, FC from caudate and pallidum seeds was measured in Service Members and Veterans with a history of mild TBI that occurred during combat deployment, Service Members and Veterans whose mild TBI occurred outside of deployment, and Service Members and Veterans who had no lifetime history of TBI.

**Results:**

FC patterns differed for the two contextual types of mild TBI. Service Members and Veterans with deployment-related mild TBI demonstrated increased FC between the right caudate and lateral occipital regions relative to both the non-deployment mild TBI and TBI-negative groups. When evaluating the association between FC from the caudate and gait, the non-deployment mild TBI group showed a significant positive relationship between walking time and FC with the frontal pole, implicated in navigational planning, whereas the deployment-related mild TBI group trended towards a greater negative association between walking time and FC within the occipital lobes, associated with visuo-spatial processing during navigation.

**Discussion:**

These findings have implications for elucidating subtle motor disruption in Service Members and Veterans with deployment-related mild TBI. Possible implications for future walking performance are discussed.

## Introduction

Since 2000, more than 450,000 Service Members have been diagnosed with traumatic brain injury (TBI), approximately 80% of which are mild in severity (1). Although a full recovery is expected, mild TBI symptoms that persist beyond the weeks and months after injury have been attributed to injury, particularly in the context of pre- or post-morbid psychiatric illness and possibly repetitive head injury (2). These symptoms (e.g., headache, memory symptoms, irritability) are also reported to persist in Veterans with mild TBI (3) who have posttraumatic stress disorder (PTSD), depression, substance use disorder, anxiety, and/ bipolar disorder which are all frequent mental health comorbidities that overlap in individuals with TBI history (4).

Recent work has investigated differences in outcomes by the context in which Service Members and Veterans (SMVs) acquire a mild TBI, specifically between SMVs who suffer a mild TBI while deployed to a combat zone (deployment TBI) and those whose injury occurred in a non-combat (non-deployment TBI) situation (5). There are a number of factors unique to a combat deployment environment that may underlie differences in outcomes, including the emotional impact, physical stress (e.g., sleep deprivation, dehydration), frequency of injuries, timing between injuries, and mechanism of injury (e.g., blunt force trauma versus blast) (6). Mild TBI acquired in a deployment environment has been associated with decrements in cognition (7), lower health-related quality of life (8, 9), and greater symptom report (8, 9). Any physiological changes unique to blast TBI may in part underlie these differences. Although they are not well characterized, greater damage to hippocampal neurons and periventricular parenchyma rather than diffuse axonal injury have been suggested (10). Recent work has also demonstrated differences in the functional connectomes of Veterans with deployment-related versus non-deployment mild TBI that may explain some differences in behavioral outcomes (11).

Veterans with repetitive blast exposure and blunt force mild TBI are reported to have significantly greater balance symptoms and higher scores on the motor scale of the Unified Parkinson’s Disease Rating Scale (UPDRS) than Veterans without blast or blunt force TBI (12), and 26.9% of Veterans with TBI due to blast report balance and coordination symptoms, compared to 4.5% of healthy control Veterans (13), suggesting there might be subtle challenges in motor activity associated with balance that negatively impact gait speed. Gait is supported, in part, by the basal ganglia, subcortical structures which include the caudate (which with the putamen and nucleus accumbens form the striatum), and the globus pallidus. Alterations in functional and structural connectivity for subcortical structures involved in motor activity such as walking have also been reported in patients with TBI (14–19).

The association between TBI and neurodegenerative disease (e.g., Parkinson’s disease) has long been a focus of the Department of Veterans Affairs (VA) and Department of Defense (DoD). Newsome et al. (15) discussed the potential relationship between altered subcortical FC and movement disorders in Veterans with deployment TBI, but did not present FC data related to motor performance. In the current study, we evaluate the relationship between FC and a measure of walking ability, gait speed, in three groups of SMVs, those who experienced mild TBI during deployment (Deployment TBI), those who experienced mild TBI outside of deployment (Non-deployment TBI), and those with entirely negative TBI history. The NIH Toolbox 4-meter walk test was included in the study because slower gait speed, beyond effects of age, is a marker of cognitive decline (20), greater risk of dementia (21–23), increased brain beta-amyloid (24), and higher risk of disability in older adults (25). We hypothesized that (1) SMVs with Deployment mild TBI would demonstrate altered FC between the basal ganglia (the caudate and globus pallidus) and occipital lobes relative to the Non-deployment mild TBI and TBI negative groups and, (2) greater FC alteration would be associated with slower walking speed.

## Methods

### Design and participants

Participants were 155 combat-exposed SMVs who were consecutively enrolled in the Long-Term Impact of Military-Relevant Brain Injury Consortium/Chronic Effects of Neurotrauma Consortium (LIMBIC/CENC) Prospective Longitudinal Study (PLS) at a single site. Participants were determined to have sustained mild TBI(s) either (1) during deployment (Deployment TBI group; *n* = 59), or (2) only outside of (i.e., prior to or following) deployment (Non-deployed TBI group; *n* = 61). A third group with a negative lifetime TBI history (TBI negative group; *n* = 35), was compared to the Deployment and Non-deployment mild TBI groups. After removal of data due to scanner artifact (*n* = 11) and excessive movement (defined as ≥50% volumes with framewise displacement greater than 0.5 mm or 3 standard deviations from the mean; *n* = 28), the final sample size was 116: Deployment TBI (*n* = 45), Non-deployment TBI (*n* = 45), and TBI negative (*n* = 26). The proportion of participants removed due to motion was highly similar across groups: 19% (Deployment TBI), 16% (Non-deployment TBI), and 20% (TBI negative). All study activities were approved by and conducted in accordance with all relevant Institutional Review Boards and other regulatory committees required by the VA and DoD. All participants provided signed informed consent prior to undergoing any study activities. Table 1 summarizes the demographic and clinical features of the groups.

**Table 1:** Sample Characteristics (*N* = 116).

	Deployment TBI (*n* = 45)		Non-Deployment TBI (*n* = 45)		Unexposed (*n* = 26)		*p*
	*M* (SD) or *n* (%)	range	*M* (SD) or *n* (%)	range	*M* (SD) or *n* (%)	range	
Age	44.29 (8.23)	30–69	43.33 (9.99)	28–61	44.96 (11.09)	25–68	0.774
Sex							0.360
Male	40 (88.89%)	─	36 (80.00%)	─	20 (76.92%)	─	
Female	5 (11.11%)	─	9 (20.00%)	─	6 (27.08%)	─	
Race							0.513
White	23 (51.11%)	─	30 (66.67%)	─	15 (57.69%)	─	
Black	20 (44.44%)	─	12 (26.67%)	─	10 (38.46%)	─	
Other	2 (4.44%)	─	3 (6.66%)	─	1 (3.85%)	─	
Ethnicity							0.520
Hispanic	2 (4.44%)	─	3 (6.67%)	─	2 (7.69%)	─	
Non-Hispanic	42 (93.33%)	─	41 (91.11%)	─	24 (92.31%)	─	
Unsure	1 (2.22%)		1 (2.22%)		0 (0.00%)	─	
Education							0.599
High School Graduate	1 (2.22%)	─	6 (13.33%)	─	4 (15.38%)	─	
Some College	21 (46.67%)	─	14 (31.11%)	─	10 (38.46%)	─	
Bachelor’s Degree	15 (33.33%)	─	15 (33.33%)	─	9 (34.62%)	─	
Master’s Degree	8 (17.78%)	─	9 (20.00%)	─	3 (11.54%)	─	
Professional Degree	0 (0.00%)	─	1 (2.22%)	─	0 (0.00%)	─	
TBI frequency	2.62 (1.51)	1–7	2.36 (1.46)	1–7	─	─	0.409
with PTA	1.20 (1.28)	0–6	1.07 (1.23)	0–6	─	─	0.624
with LOC	1.33 (1.28)	0–6	0.51 (0.73)	0–3	─	─	<0.001
Years since most recent TBI	12.41 (9.84)	0.32–43.66	15.13 (11.04)	0.90–45.24	─	─	0.221
PCL-5 Total Score	25.62 (20.34)	1–66	19.27 (15.39)	0–57	3.35 (2.88)	0–11	<0.001
PHQ-9 Total Score	7.57 (6.16)	0–23	6.45 (6.04)	0–21	13.42 (11.69)	0–41	<0.001
Gait Speed Score	1.28 (0.23)	0.62–1.80	1.29 (0.19)	0.88–1.75	1.28 (0.20)	1.10–1.85	0.969

LOC = Loss of Consciousness, TBI = traumatic brain injury, PCL-5 = Posttraumatic Stress Disorder (PTSD) Checklist for DSM-5, PHQ-9 = Patient Health Questionnaire-9, PTA = Posttraumatic Amnesia, SD = Standard Deviation. Group comparison *p*-values were calculated with ANOVA, *t*-tests, and chi-square as appropriate for data type.

### Procedures

#### Behavioral measures

Behavioral measures were collected as part of the larger LIMBIC-CENC PLS battery. The PTSD Checklist for The Diagnostic and Statistical Manual of Mental Disorders, Fifth Edition (PCL-5) is a 20-item self-report measure of four clusters of PTSD (intrusion, avoidance, negative alterations in cognitions and mood, and alterations in arousal and reactivity) (26). Higher scores indicate greater symptom severity. In Veterans, the PCL-5 has excellent internal consistency (Cronbach’s α = 0.96) and test–retest reliability (*r* = 0.84) (27).

The 9-Item Patient Health Questionnaire (PHQ-9) (28) is a self-report measure of depression. Higher numbers indicate greater severity. Internal (Cronbach’s α = 0.89) and test–retest reliability (Cronbach’s α = 0.83) are excellent.

Gait speed is an important aspect of motor performance and was measured with the NIH Toolbox 4-meter walk test (29). Normative values are available for the test. The intraclass correlation coefficients are considered fair (0.41–0.46) (29). Participants are asked to walk 4 meters at their usual pace, and the time in seconds is measured during each of two trials, with the shortest time used for analysis. A score of meters per second is calculated by dividing 4 by the number of seconds. Higher numbers indicate slower speeds. As a point of reference, the mean gait speed score of a community-dwelling sample of males aged 30–49 years is 1.21; for females 30–49 years, the mean gait score is 1.15 (29).

#### Functional connectivity

During the resting state acquisition, the MRI technologist instructed each participant to lie still with eyes open and fixated on a marker at the top of the bore and comfortably within their line of sight. MRI technologists spoke with subjects immediately before and after the resting state sequence to provide instructions and to ascertain wakefulness and recorded the information on a form. None of the participants included in the analysis were determined to have fallen asleep during the imaging session.

#### Image data acquisition

Whole brain imaging was performed using a 32-channel head coil on a Philips 3 T Ingenia system (Philips, Best, Netherlands) at the Collaborative Advanced Research Imaging facility (CARI), Wright Center for Clinical and Translational Research, Virginia Commonwealth University. Regular quality assurance (QA) testing that included QA monitoring of EPI stability (30) as well as geometric accuracy (31) was performed throughout the course of the study, and no issues were detected. BOLD T2*-weighted echo-planar images (EPI) were acquired as 200 volumes with 48 contiguous axial slices of 3.3 millimeter (mm) thickness, 212-mm field of view (FOV), 64 × 64 matrix, repetition time (TR) of 3,000 ms, echo time (TE) of 30 ms, and an 80-degree flip angle. A set of three dimensional (3D) high-resolution T1-weighted images were also acquired in 170 sagittal slices of 1.2 mm thickness (no gap) with 240 mm FOV, 256 × 256 matrix, TR of 6.78 ms, TE of 3.16 ms, and a 9.0-degree flip angle.

### Statistical analysis

#### Demographic and behavioral data

Characteristics of the sample are summarized in means, standard deviations, and ranges for continuous variables and counts and percentages for categorical variables. Chi-square tests were performed for categorical comparisons, t-tests for comparisons with two groups, and ANOVA for three groups.

#### Functional connectivity image processing and analysis

The Functional Connectivity Toolbox (Conn) (32) within Statistical Parametric Mapping (SPM) SPM8 (Wellcome Department of Cognitive Neurology, University College, London, UK) implemented in Matlab (Mathworks Inc. Sherborn MA, USA) was used to process and analyze data. Functional images of each participant were realigned, co-registered with each participant’s high resolution anatomical image, normalized to the Montreal Neurological Institute (MNI) template, and smoothed using a 6 mm Full Width - Half Maximum (FWHM) Gaussian filter. Anatomical landmarks in the normalized high resolution anatomical and functional data were visually checked and compared against the MNI template for each participant. Each participant’s anatomical image was segmented into gray matter (GM), white matter (WM) and cerebrospinal fluid (CSF) masks. Physiological noise was addressed by using average activity within the WM and CSF masks as covariates. Realignment parameters and their first-order derivatives were also covaried. To repair artifact due to frame-by-frame head movement, outlier time points were defined as exceeding 0.5 mm or three standard deviations from the mean image intensity of the complete resting state run, and outliers were included as regressors in the first level general linear model along with motion parameters and their first-order derivatives. Data were band-pass filtered between 0.008 and 0.09 Hz, the default frequency range in the SPM Conn toolbox. The high-pass value was selected to approximate both SPM’s default value (0.0078 Hz) and a two-minute value suggested as a standard (0.0083 Hz) (33). The low-pass value approximates the frequently reported 0.08 Hz and 0.10 Hz values and SPM’s hemodynamic response function cutoff frequency of 0.091 Hz. FC was measured with single seeds in the left and right caudate and left and right pallidum anatomically defined in the FSL-Harvard Atlas.

A general linear model was used to estimate the correlation between the seeds and the whole brain on a voxel-wise level for individual participants in a first level analysis. Pearson correlation coefficients were then transformed into z-scores using Fisher’s method. Group (second level) whole-brain voxel-wise random effects analyses were conducted using the general linear model, in which t-tests were calculated for planned comparisons between the two TBI groups and for each TBI group compared to the TBI negative control group. Analysis of covariance (ANCOVA) with age and total scores on the PHQ-9 and PCL-5 as covariates was then performed to investigate whole brain voxel-wise differences in FC between TBI groups and for each TBI group compared to the TBI Negative group. We first performed simple regressions of age and PHQ-9 and PCL-5 total scores onto the FC of each seed from each group. If results were not significant, the variable was not entered into an ANCOVA. Each covariate was entered separately after verifying the homogeneity of slopes assumption for each seed on a voxel-wise basis.

Regression analysis was conducted for the mean-centered gait speed scores of each subject onto the z-scores representing FC within each group. The regression slopes were then compared between the two TBI groups. The TBI negative group was not included in the comparison between regression slopes because gait speed scores were available for only a subset (*n* = 16) of these subjects.

In SPM, a cluster of voxels is defined as a set of voxels that survives a cluster-defining voxel (height) threshold and which occur spatially contiguous with each other. In this study, the cluster-defining height threshold was set at *p* < 0.001, uncorrected, recommended for control of inflated cluster extent (34). Significance at the cluster level of inference was defined by a corrected cluster threshold of *p* < 0.05, after False Discovery Rate (FDR) correction for multiple comparisons across the whole brain. Further Bonferroni correction was made for the number of seeds, groups, and tails, or directions, (criterion *p* = 0.05/[4 seeds × 3 groups × 2 directions] = *p* = 0.002).

## Results

### Symptom and demographic measures

See Table 1. Most group comparisons were nonsignificant, including gait scores, which did not differ among the three groups. The TBI negative group reported significantly lower PCL-5 and PHQ-9 scores than either TBI group, and the Deployment TBI group reported more TBIs with loss of consciousness (LOC) than the Non-deployment group.

### Functional connectivity group differences

Results for all FC between-group differences are reported in Table 2. Analyses including covariates are reported in the Supplemental Information (SI; Supplementary Table 1).

**Table 2:** Between Group Analyses Evaluating Left and Right Caudate and Globus Pallidus Seeds.

Group comparison	Cluster-level *p* value (corrected)^a^	Cluster size (k)^b^	Most significant coordinates^c^			Location
			x	y	z	
Deployed > Nondeployed						
a. Left caudate seed	<0.000049	555	64	−50	34	R superior lateral occipital cortex, angular gyrus
b. Right caudate seed	*ns*					
c. Left globus pallidus	*ns*					
d. Right globus pallidus	*ns*					
Nondeployed > Deployed	*NS*					
Deployed > Unexposed						
a. Left caudate seed	< 0.000033	570	−42	−76	38	R superior lateral occipital cortex, angular gyrus
	< 0.000033	562	4	−52	20	Precuneus, PCC
b. Right caudate seed	*ns*					
c. Left globus pallidus	*ns*					
d. Right globus pallidus	*ns*					
Unexposed > Deployed						
a. Left caudate seed	*ns*					
b. Right caudate seed	*ns*					
c. Left globus pallidus	*ns*					
d. Right globus pallidus	<0.00484^*^	253	−4	−6	46	Bilateral precentral gyri, juxtapositional cortices (formerly SMA), ACC
Nondeployed > Unexposed	*NS*					
Unexposed > Nondeployed	*NS*					

a Probability at the cluster level of significance after random field theory family-wise error correction over the whole brain search volume. Cluster probability also survives Bonferroni correction for number of seeds, groups, and tests, (criterion *p* = 0.05/[4 seeds × 3 groups × 2 types of test] = *p* = 0.002).

b Number of voxels within a cluster.

c Negative values along the x-axis are defined to be in the subject’s left hemisphere.

* Marginal significance.

#### Deployment vs. non-deployment TBI

##### Deployment > Non-deployment TBI

**Figure 1:**
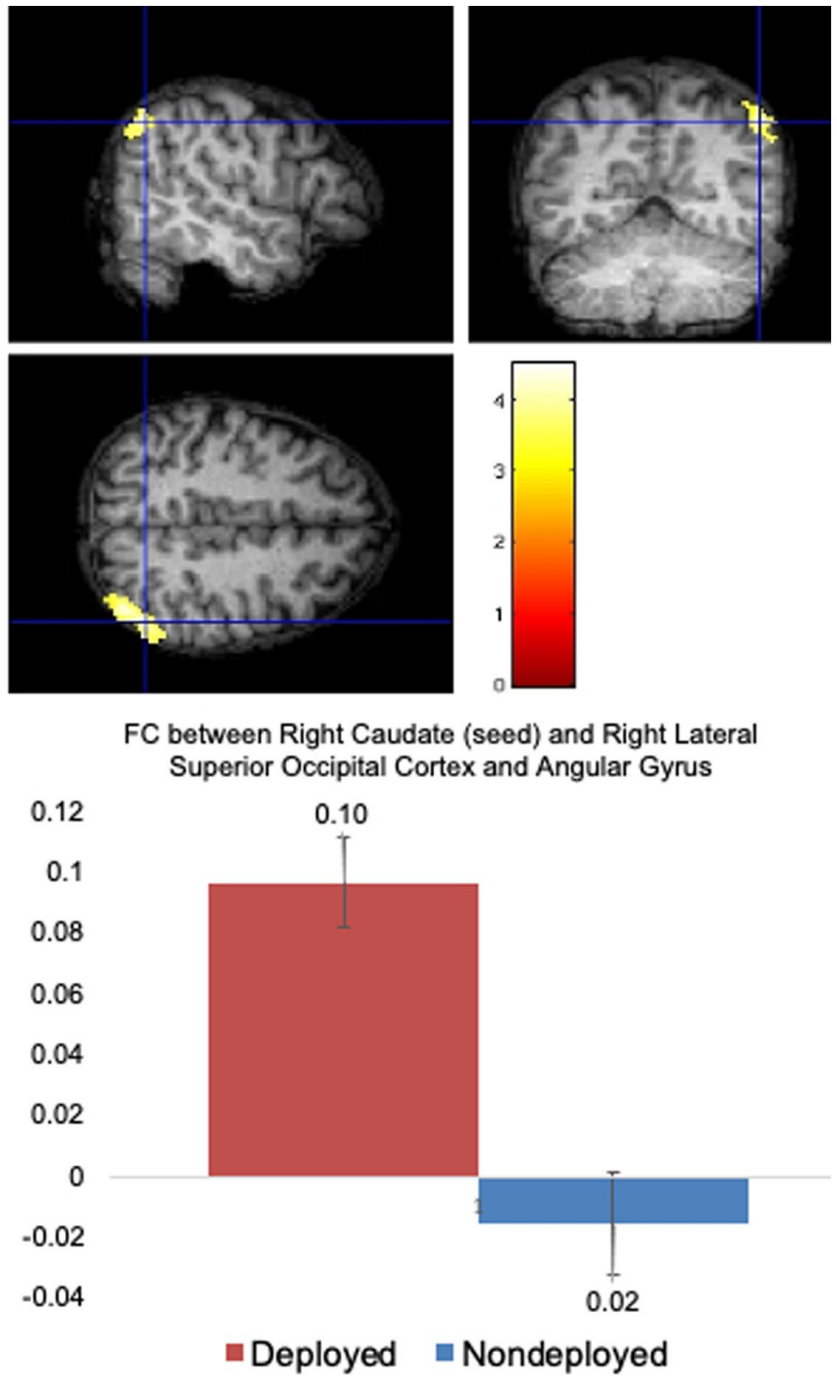
Deployed > Non-deployed. The Deployed mTBI group demonstrated significantly greater FC between right caudate (seed) and right lateral superior occipital cortex and angular gyrus than the Nondeployed group. Right side of brain is on the right side of the screen. Color in bar reflects *t*-value. Columns in the bar chart depict means and standard errors.

Compared to the Non-deployment TBI group, the Deployment TBI group demonstrated greater FC between the right caudate seed and one cluster in the right superior lateral occipital cortex and angular gyrus (FDR and Bonferroni-corrected cluster threshold *p* < 0.000049, FDR corrected, beta = 0.11, 90% CI [0.08, 0.14]), which remained significant when covarying PHQ-9 and PCL-5 total scores (see Figure 1, Table 2, and Supplementary Table 1). No other seeds were significant.

##### Non-deployment > Deployment TBI

The Non-deployment TBI group did not have any FC that was significantly greater than the Deployment TBI group.

#### Deployment TBI vs. TBI negative

##### Deployment TBI > TBI negative

Compared to the TBI negative group, the Deployment TBI group demonstrated greater FC between the left caudate seed and one cluster in the right superior lateral occipital cortex and angular gyrus (cluster threshold *p* < 0.000033, FDR corrected, beta = 0.15, 90% CI [0.01, 0.19]), and one cluster in precuneus and posterior cingulate cortex (PCC; cluster threshold *p* < 0.000033, FDR corrected, beta = 0.14, 90% CI [0.08, 0.19]), which remained significant when covarying PHQ-9 and PCL-5 total scores (see Table 2 and Supplementary Table 1).

##### TBI negative > Deployment TBI

The TBI negative group showed greater FC between the right pallidum seed and a cluster in bilateral precentral gyri, juxtapositional cortices (formerly supplementary motor cortex), and anterior cingulate gyrus that approached significance (cluster threshold *p* < 0.004084, FDR corrected, beta = 0.12, 90% CI [0.07, 0.17]) and met significance when covarying PHQ-9 and PCL-5 total scores (see Table 2 and Supplementary Table 1). No other seeds were significant.

#### Non-deployment TBI vs. TBI negative

No seeds were significant.

### Functional connectivity regressions of gait speed scores onto FC

The TBI negative group was examined separately due to smaller sample size to better understand regions associated with walking performance in the absence of mild TBI history.

#### TBI negative

See Table 3. Positive correlation was nonsignificant for the TBI negative group. However, this group demonstrated a significant negative correlation between gait scores and FC between the left caudate and precuneus (cluster threshold *p* = 0.001073 FDR corrected, beta = −0.65, 90% CI [−0.85, −0.48]), which was also significant after covarying PHQ-9 total scores, but only trending after covarying PCL5 total scores. Covarying PHQ-9 total scores revealed an additional significant cluster in the left temporal pole, anterior inferior temporal gyrus, and anterior temporal fusiform gyrus, which was marginally significant after covarying PCL-5 total scores. No other seeds were significant.

**Table 3:** Regression analyses relating walk time onto the functional connectivity of caudate and globus pallidus seeds.

	Cluster-level *p* value (corrected)^a^	Cluster size (k)^b^	Most significant coordinates^c^			Location
			x	y	z	
Unexposed only						
a. Left caudate						
Positive Regression	*ns*					
Negative Regression	<0.001073	199	6	−54	40	Precuneus
b. Right caudate	*ns*					
c. Left globus pallidus	*ns*					
d. Right globus pallidus	*ns*					
Deployed > Nondeployed						
a. Left caudate	*ns*					
b. Right caudate						
Positive Regression	*ns*					
Negative Regression	<0.0000001	1720	−10	−48	8	Precuneus, Posterior Cingulate Gyrus, Bilateral Lingual Gyrus
	<0.000193	404	58	−4	−28	R Middle Temporal Gyrus, Right Temporal Pole, R Inferior Temporal Gyrus
	<0.000321	357	50	−66	24	R Superior Lateral Occipital Cortex
c. Left globus pallidus	*ns*					
d. Right globus pallidus	*ns*					
Nondeployed > Deployed						
a. Left caudate	*ns*					
b. Right caudate						
Positive regression	<0.001	585	38	42	26	Frontal Pole (middle frontal gyrus)
Negative Regression	*ns*					
c. Left globus pallidus	*ns*					
d. Right globus pallidus	*ns*					

a Probability at the cluster level of significance after random field theory family-wise error correction over the whole brain search volume. Cluster probability also survives Bonferroni correction for number of seeds, groups, and tests, (criterion *p* = 0.05/[4 seeds × 3 groups × 2 types of test] = *p* = 0.002).

b Number of voxels within a cluster.

c Negative values along the x-axis are defined to be in the subject’s left hemisphere.

#### Deployment vs. non-deployment TBI

##### Deployment > Non-deployment

**Figure 2:**
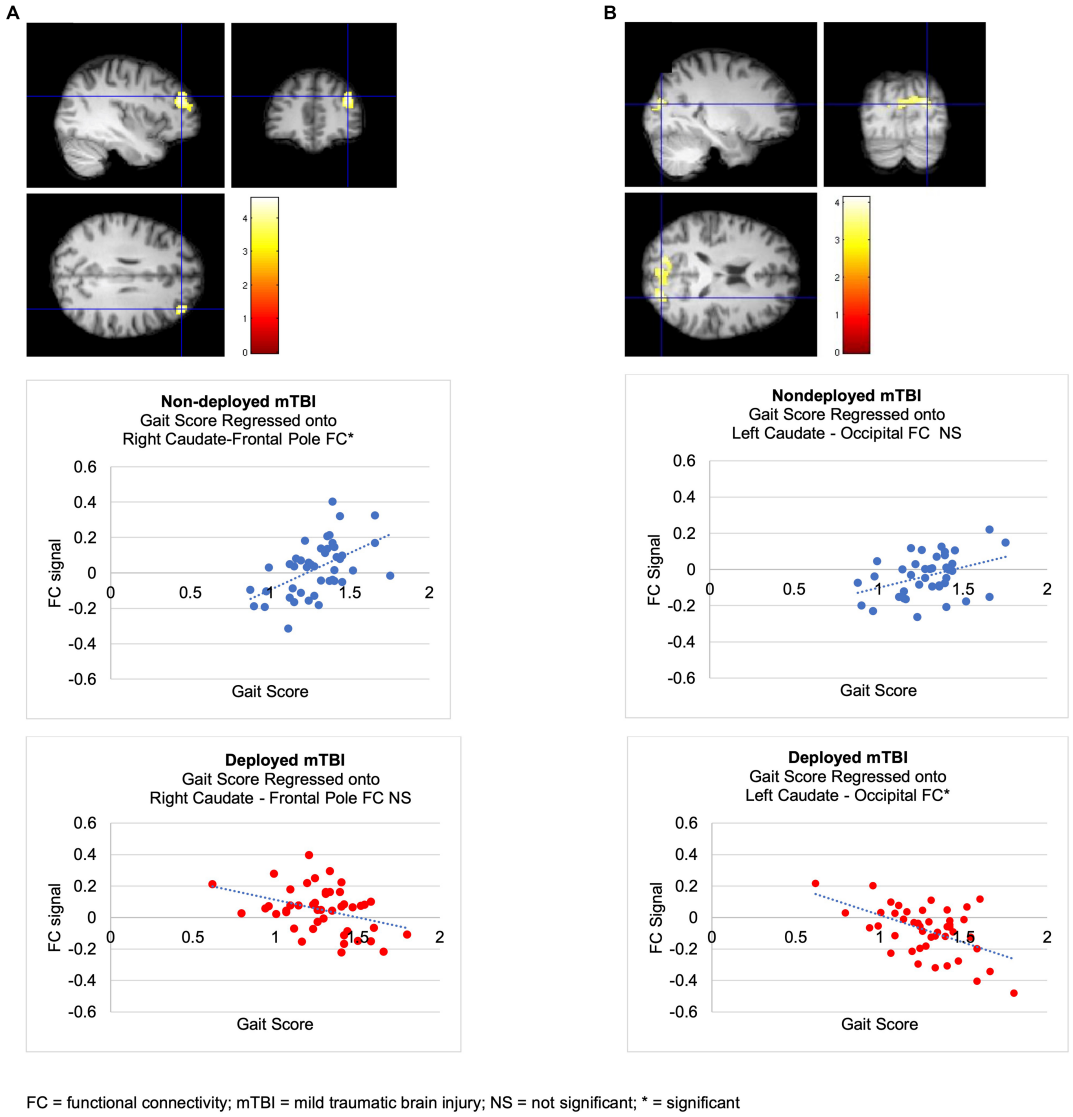
Regressions of walk scores onto caudate FC in mTBI groups. **(A)** Non-deployed > Deployed. The Non-deployed mTBI group demonstrated a significantly greater positive relation between walk scores and FC between the right caudate (seed) and right frontal pole than the Deployed mTBI group. Examining regressions of each group separately confirmed that the significant multiple regression with group was due to a positive regression between walk scores and right caudate-right FP FC in the Non-deployed group and revealed no significant clusters in the Deployed group. **(B)** Deployed > Non-deployed. The Deployed mTBI group demonstrated trends toward a greater negative relation between walk scores and FC between the left caudate and two occipital clusters (*p*s = 0.005107 and 0.007525). Examining regressions of each group separately confirmed the negative regressions between walk scores and left caudate-occipital FC in the Deployed group and revealed no significant clusters in the Nondeployed group.

The Deployment TBI group demonstrated greater negative correlations between gait scores and left caudate FC that did not survive the Bonferroni correction for multiple seeds, groups, and tails (*p* < 0.002), with two clusters involving the right occipital fusiform gyrus, lingual gyrus, and cerebellum VI, a lobule of the posterior lobe (Cluster 1, cluster threshold *p* = 0.005, FDR corrected, beta = 0.57, 90% CI [0.38, 0.77]), and the right superior lateral occipital cortex and occipital pole, and bilateral cuneus (Cluster 2, cluster threshold *p* = 0.008, FDR corrected, beta = 0.60, 90% CI [0.37 0.88]). Examining regressions of each group separately confirmed the negative regressions between walk scores and the two clusters in the left caudate-occipital FC in the Deployment TBI group and no significant clusters in the Non-deployment TBI group. Covarying for PCL-5 and PHQ-9 total scores revealed similar clusters that were marginal in significance, *p*s ≤ 0.010 (see Figure 2 and Table 3). No other seeds were significant.

##### Non-deployment > Deployment TBI

The Non-deployment mild TBI group demonstrated a greater positive association than the Deployed group between gait scores and FC between the right caudate and right frontal pole (cluster threshold *p* = 0.001 FDR corrected, beta = 0.64, 90% CI [0.43, 0.85]), which was also significant or approached significance after covarying PCL-5 and PHQ-9 total scores (cluster threshold *p* = 0.002, and *p* = 0.003, FDR corrected, respectively; see Figure 2 and Table 3).

## Discussion

We investigated the effect of mild TBI acquired during military combat deployment on subcortical brain structures associated with movement. SMVs who sustained mild TBI during deployment showed increased FC between the right caudate seed and superior lateral occipital cortex and angular gyrus compared to SMVs with non-deployment mild TBI, and SMVs who are TBI negative, suggesting some robustness to the pattern of subcortico-occipital FC specific to combat deployed SMVs with mild TBI. We also observed that altered FC was related to a gait measure. The Non-deployment mild TBI group demonstrated a greater positive association when walk scores were regressed onto FC between the right caudate seed and the right frontal pole than did the Deployment TBI group. Conversely, compared to the Non-deployment group, the Deployment TBI group demonstrated a marginally significant greater negative association between left caudate and occipital regions also including the superior lateral occipital cortex, as well as the lingual gyrus, occipital fusiform gyrus, cerebellum VI, cuneus, and occipital pole. They showed the same pattern when compared to the TBI negative comparison group. FC between the caudate and occipital lobes appears to occur when TBI is acquired during combat deployment and is related to the speed with which combat SMVs with mild TBI history walk.

The regions identified through regression analysis in the Deployment TBI group compared to the TBI negative group and were associated with gait speed are similar to those previously reported (15) (occipital fusiform gyrus, lingual gyrus, cerebellum VI, cuneus, and occipital pole), suggesting that the previous results might have been linked with gait speed. However, there are some differences between the two studies. In Newsome et al. (15), the globus pallidus, rather than the caudate, was significantly correlated with occipital regions. Additionally, the altered FC between the globus pallidus and occipital lobes was an increased anti-correlation rather than the increased positive FC found in this analysis (i.e., above zero in the graph in Figure 1). The proximity of the globus pallidus to the caudate, the imprecise nature of blast impacts, different post-injury intervals [i.e., 5.46 years (15) versus 12.41 years in the current study], and different comparison groups might contribute to differences.

Why would the occipital cortex demonstrate increased FC with the basal ganglia? Occipital cortex and basal ganglia have been reported to be anatomically connected and show co-activation during task and resting state fMRI (rsfMRI) (35–38). In a diffusion study mapping the basal ganglia connectome, lateral occipital cortex was shown to have weak connectivity with globus pallidus and striatum, albeit the putamen (35). In a follow-up study investigating topographical organization as part of the Human Connectome Project, connectivity was found between the globus pallidus and occipital and other lobes (36). In a meta-analysis on human navigation, right caudate was implicated in navigation when objects in a room were understood in relation to a walker’s position (egocentric), but not when a walker’s position was not linked to objects in a room (allocentric) (37). Both types of navigation involve mental imagery and were linked to activation in fusiform and lingual gyri, precuneus, cuneus, and middle frontal gyrus (37), regions observed in the current study. Intriguingly, FC between basal ganglia and occipital lobes was increased in patients with a movement disorder, essential tremor. The patients demonstrated increased FC between extrastriate cortex (which includes lingual gyrus, cuneus, and superior occipital gyrus) and basal ganglia (globus pallidus, caudate, putamen) compared to healthy controls (38). The authors attributed the increased FC to enhanced visual feedback (i.e., seeing the tremor themselves) compared to the healthy controls.

Although our participants with Deployment TBI were not given visual feedback, they may have relied more than the other groups on visual information while navigating the room. In a meta-analysis of mapping brain regions to cognitive tasks, the superior lateral occipital cortex was linked to visuospatial tasks and other tasks involving viewing motion (39). The angular gyrus, identified along with the superior lateral occipital cortex to demonstrate increased FC in the Deployment TBI group, has been implicated in identification of one’s location in space and time (40).

Overall gait speed means and variances of the three groups were similar, while the relationships between the gait speed scores and FC differed, suggesting that gait itself is not impaired, but the regions each group relied on for gait may be linked to the environment in which the injury occurred. Possibly the extra-FC is required to yield the same results (i.e., support gait) as in non-deployed individuals. In Veterans with mTBI, group differences in functional brain imaging have been reported when there were no group differences in performance of a task (41). Additionally, areas that are responsible for performing a task in healthy people and show altered FC in TBI patients may or may not demonstrate reliability over time. If the altered FC pattern is not available at a later time point, it may not be sufficient for supporting ongoing task performance.

Speculatively, the FC of the Deployment TBI group may reflect individuals’ utilizing visuospatial cues in their environment more than the Non-deployment mild TBI or TBI negative groups did. It is also possible that regions for visuospatial processing adapt to provide other types of processing related to walking. Non-deployment TBI, on the other hand, revealed a positive relation between gait speed and FC between right caudate and right frontal pole (i.e., middle frontal gyrus/dorsolateral prefrontal cortex). In a meta-analysis, this region has been implicated specifically in planning while navigating (37). Individuals with non-deployment TBI may rely more on planning when walking is slowed, whereas individuals with deployment TBI may rely more on visuospatial processing, which becomes less accessible for slower gaits given the negative regression between FC and gait speed scores. If this pattern replicates in larger samples, to better understand gate mechanisms, an intriguing follow-up line of inquiry would investigate any pattern in regions the SMVs at the upper end of the range (the slower walkers) recruit and the cognitive processes they employ to potentially guide future rehabilitation in gait-cognition coupling.

In the between-group comparisons, the TBI negative group, compared to the Deployment TBI group, demonstrated greater FC between the caudate and juxtapositional cortex (formerly supplementary motor area [SMA]) than the TBI negative group. When evaluating the association between FC and walking scores, the TBI negative group demonstrated a positive association with the precuneus. The SMA is directly involved in walking and is connected to the caudate and putamen via the frontostriatal tract (42). It is possible that this tract is disrupted in Veterans with deployment TBI, potentially causing them to rely more on other tract(s) to ensure a connection between the caudate and occipital lobes.

The TBI negative group also demonstrated a significant negative association between walk scores and FC between the caudate and precuneus, suggesting that the precuneus plays an important role in walking, particularly faster gait, for SMVs without TBI. The precuneus is anatomically and functionally connected to the caudate (43, 44), and shares FC with the motor and supplementary motor cortices in healthy adults (44), supporting its role in walking.

The pattern of increased FC seen in both TBI groups resembles the hyperconnectivity and hyperactivation often seen in individuals with TBI and has been attributed to additional regions compensating for impaired ones. Hyper-FC often occurs in regions that are recruited in healthy participants as well as other regions. In the analysis evaluating walking scores, the TBI negative group demonstrated greater FC in the precuneus, also known to be involved in walking in healthy non-SMVs. The precuneus showed increased FC in the Deployment TBI group in addition to neighboring and other posterior regions. Many brain regions can be classified with subcomponent regions, and it is possible that parts of the regions providing the compensation are themselves compromised. In that scenario, other regions are recruited because one is not sufficient. Many of the regions noted in our study as having increased FC in the Deployment TBI group are also reported as having altered glucose metabolism in Veterans with mild TBI (12), suggesting they may not be fully functioning.

Compensatory reliance on brain regions not typically associated with gait could precede potential walking difficulties after mild TBI if the altered FC is not reliable or able to provide the neural support necessary for healthy gait, a topic of a potential longitudinal study for future research.

Strengths of the study include the following: (1) Use of study participants at a single site using the same scanner, which eliminated noise that can be incompletely controlled for when using multiple sites and scanners; (2) Inclusion of three groups of SMVs allowing us to compare two types of TBI context to each other and to controls; (3) Requirement that control subjects (TBI-negative group) also have a history of combat deployments. It is often not possible to recruit control subjects with backgrounds similar to the patient population; (4) Use of validated interview methods (rather than self-report) to determine lifetime mTBI histories in all subjects by using validated structured interviews followed by local site review by the principal investigator, as well as vetting to confirm a computer algorithm diagnosis of mTBI for every potential concussive event and a centralized expert committee that adjudicates any remaining uncertain mTBI diagnoses; (5) finally, another strength is that by comparing gait speed, or other aspects of walking, with basal ganglia connectivity, we may be able to define novel endophenotypes that, with further study, may guide clinical care. There are also several limitations in this study. Bonferroni correction was calculated for the number of seeds, groups, and tails, but not for the number of tests (t-tests and ANCOVAs). A follow-up study with increased sample sizes will be more equipped to handle the conservative nature of Bonferroni correction. The PCL-5 and PHQ-9 have some overlap in the symptoms they measure and might have led to some degree of redundancy in the results of the ANCOVAs; however, results of between-group tests with and without covariates were similar, suggesting that both measures might not have significant effects on the results. The PCL-5 does not provide as detailed an assessment of PTSD symptoms and their clinical effect as the Clinician Administered PTSD Scale (CAPS-5) (45). FC from the putamen, a prominent brain region in the basal ganglia was not measured; however current seeds were based on the results of Newsome et al. (15). FC was not measured during the performance of an imagined walking test in the scanner; brain regions involved in walking imagery tasks have been shown to closely parallel those involved in actual walking in healthy adults, and a walking imagery task is feasible in patients with severe TBI (46). Lastly, the TBI groups were closely matched, except the Deployed group had significantly more TBIs with loss of consciousness (LOC) than the Non-deployed group. LOC in Veterans has been known to be related to altered WM in the brainstem (47). Interestingly, however, subjects with severe TBI who also had disorders of consciousness acutely demonstrated altered default mode network (DMN) functional connectivity while they were comatose, but 6 months later during recovery, their DMN patterns were “indistinguishable” from those of healthy adults (48). While this finding strongly suggests that LOC does not alter the DMN, other FC networks were not tested.

Mild TBI in Veterans has been linked to dementia (49), Parkinson’s disease (PD) (50), and progressive neurodegeneration as measured by retinal thickness (51). Slowing in gait speed is associated with risk of dementia and PD in non-SMVs. Although overall gait scores did not differ between groups in the present study, increased FC was associated with faster gait, suggesting that hyper-FC may assist in faster gait. It is possible that people with slower gait were not as adept at functionally connecting the caudate and occipital areas. Alternatively, it is possible that improvements in gait speed scores could be linked to reductions in hyper-FC. In addition to further study of features of walking other than gait speed, such as balance and coordination, patients with hyper-FC might benefit from treadmill therapy to strengthen connections. Treadmill therapy, i.e., a six-week intervention of increasing pace on a treadmill as tolerated, combined with a virtual reality cognitive component, is associated with increased gait speed, improved balance and reduction of FC from the striatum in PD patients (52), and may be useful in Veterans with mild TBI as preventative or early treatment. Further, aberrant FC may precede aberration in behavior. To better understand if deployment-related TBI uniquely impacts FC-gait coupling, future longitudinal studies may test for shifts in compensatory patterns over time in TBI of different etiologies and relate them to performance to potentially provide predictive biomarkers for changes in gait. Furthermore, future rehabilitation clinical trials may benefit by using these novel biomarkers for patient stratification, reducing noise in a complex patient population.

## Conclusion

When investigating two etiological settings for mild TBI, we found that SMVs who incurred mild TBI during combat deployment demonstrated increased FC between the basal ganglia and occipital lobes compared to SMVs whose mild TBI occurred outside of deployment and to SMVs who did not have mild TBI. The superior lateral occipital cortex was implicated in the Deployment TBI group both in the between group comparisons and in a negative regression of walking scores onto FC, suggesting a reliance in the Deployment mild TBI group on areas involved in navigation that becomes less available when walking speed is slower. The Non-deployment TBI group demonstrated a positive relationship between walk scores and FC between the caudate and frontal pole, involved in planning during navigation. Findings have implications for elucidating subtle motor disruption in two types of mTBI in SMVs; despite intact walking performance, changes in FC occur, which could have implications for future walking performance.

## Data availability statement

Data will be made available to researchers after they have submitted a proposal to the LIMBIC CENC Consortium, it has been approved, and all necessary data sharing agreements have been executed. For further information kindly refer to the LIMBIC-CENC webpage, https://www.limbic-cenc.org/for-tbi-researchers/data-requests-public/.

## Ethics statement

The studies involving humans were approved by Richmond Institute for Veterans Research (formerly known as McGuire Research Institute, Inc.). The studies were conducted in accordance with the local legislation and institutional requirements. The participants provided their written informed consent to participate in this study.

## Author contributions

MN: Conceptualization, Formal analysis, Investigation, Methodology, Writing – original draft, Writing – review & editing. SM: Writing – original draft, Writing – review & editing. ND: Writing – review & editing. ED: Writing – review & editing. MD: Data curation, Writing – review & editing. CE: Writing – review & editing. CH: Writing – review & editing. GJ: Writing – review & editing. QL: Data curation, Formal Analysis, Writing – review & editing. KK: Writing – review & editing. AM: Writing – review & editing. JR: Writing – review & editing. RS: Writing – review & editing. JS: Writing – review & editing, Formal Analysis, Methodology. BT: Writing – review & editing, Data curation. JW: Writing – review & editing. DT: Writing – review & editing. WW: Funding acquisition, Project administration, Writing – review & editing. EW: Writing – review & editing, Funding acquisition, Project administration, Resources.
