# Supplementary material for: Subcortical functional connectivity and its association with walking performance following deployment related mild TBI
Source: Front Neurol. 2023 Dec 14;14:1276437. doi: 10.3389/fneur.2023.1276437 (PMC10752967; doi:10.3389/fneur.2023.1276437)
Supplement: Supplementary file 1 [file Table_1.DOCX]

Supplementary Table 1. Results from between group analyses with and without using PHQ-9 and PCL5 Total scores as separate covariates. PHQ-9 and PCL5 were entered separately as some items might measure similar constructs. Caudate and globus pallidus seeds are listed with any significant clusters.

1. Deployed > Nondeployed

a. Left caudate seed

NS

b. Right caudate seed

Cluster-Level Cluster Most Significant

Covariate *p* Value Size (k)^b^ Coordinates^c^ Location

(corrected) ^a^ (x y z)

____________________________________________________________________________

*n/a* 0.000049 555 64 -50 34 R sup lateral occ

cortex

R angular gyrus

PHQ-9 0.000063 543 64 -50 36 R sup lateral occ

cortex

R angular gyrus

PCL5 0.000129 491 66 -50 34 R sup lateral occ

cortex

R angular gyrus

c. Left globus pallidus

NS

d. Right globus pallidus

NS

*2.* Nondeployed > Deployed

All seeds NS

*3.* Deployed > Unexposed

a. Left caudate seed

Cluster-Level Cluster Most Significant

Covariate *p* Value Size (k)^b^ Coordinates^c^ Location

(corrected) ^a^ (x y z)

____________________________________________________________________________

*n/a* *p* < 0.000033 570 -42 -76 38 R sup lateral

occ cortex,

R ang gyr

*p* < 0.000033 562 4 -52 20 Precuneus,

PCC

PHQ-9 *p* < 0.000020 614 -42 -76 38 R sup lateral

occ cortex

*p* < 0.000044 523 4 -52 20 Precuneus, PCC

PCL5 *p* < 0.000026 601 -40 -76 40 R sup lateral

occ cortex

*p* < 0.000049 519 4 -52 20 Precuneus, PCC

b. Right caudate seed

NS

c. Left globus pallidus

NS

d. Right globus pallidus

NS

4. Unexposed > Deployed

a. Left caudate seed

NS

b. Right caudate seed

NS

c. Left globus pallidus

NS

d. Right globus pallidus

Cluster-Level Cluster Most Significant

Covariate *p* Value Size (k)^b^ Coordinates^c^ Location

(corrected) ^a^ (x y z)

____________________________________________________________________________

*n/a* *p* < 0.00484* 253 -4 -6 46 Bilateral precentral gyri,

Juxtapositional cortices (formerly SMA),

ACC

PHQ-9 *p* < 0.00213 294 -4 -6 46 Bilateral precentral gyri,

Juxtapositional cortices

(formerly SMA),

ACC

PCL5 *p* < 0.001198 314 -4 -6 46 Bilateral precentral gyri,

Juxtapositional cortices

(formerly SMA),

ACC

5. Nondeployed > Unexposed

All seeds NS

6. Unexposed > Nondeployed

All seeds NS

_____________________________________________________________________________________

* = Marginally significant, ACC = anterior cingulate cortex, n/a = not applicable, NS = nonsignificant, Occ = occipital, PCC = posterior cingulate cortex, PCL5 = PTSD Checklist for DSM V, PHQ-9 = Patient Health Questionnaire – 9, R = right, SMA = supplementary motor area, Sup = superior
